# Histogenesis of the Uterine Horn in the Domestic Cat (*Felis silvestris catus*): LM, TEM, and SEM Study

**DOI:** 10.3390/ani15142067

**Published:** 2025-07-13

**Authors:** Ewelina Prozorowska-Basińska, Marlena Ratajczak, Hanna Jackowiak

**Affiliations:** 1 Department of Histology and Embryology, Faculty of Veterinary Medicine and Animal Science, Poznan University of Life Sciences, Wojska Polskiego 71C, PL-60-625 Poznan, Poland; hanna.jackowiak@up.poznan.pl; 2 Laboratory of Electron and Confocal Microscopy, Adam Mickiewicz University, Uniwersytetu Poznanskiego 6, PL-61-614 Poznan, Poland; marlena.ratajczak@amu.edu.pl

**Keywords:** uterus, histogenesis, domestic cat, prenatal development

## Abstract

This study employs light microscopy, scanning electron microscopy, and transmission electron microscopy to describe the morphological changes occurring during the development of the domestic cat’s uterine horns, originating from the uterine segments of paramesonephric ducts (uPD). Comprehensive observations conducted on 60 specimens aged 28–63 days post-conception (p.c.) revealed that the formation of the endometrium and myometrium in the uterine horns begins around day 33 p.c., initiated by mesenchymal differentiation. During endometrial development, fibroblasts align first in perpendicular and then in oblique columns. The subdivision of the lamina propria into basal and functional layers becomes evident shortly before birth, with the functional layer remaining flat until the end of the prenatal period. The endometrial epithelium transforms from a simple columnar to a pseudostratified structure, undulating by day 63 p.c. Myometrial formation commences with the differentiation of myoblasts, which are arranged in a circular pattern. By the end of gestation, these myoblasts differentiate into smooth muscle cells, organizing into distinct inner circular and outer longitudinal sublayers. Although the fundamental layered architecture of the uterine wall is established before birth, its full maturation—including gland formation, epithelial transformation, and further development of the myometrium—continues postnatally.

## Simple Summary

This study investigates the prenatal development of the domestic cat’s uterine horns, originating from the paramesonephric (Müllerian) ducts. The aim was to describe the histodifferentiation of the uterine wall, focusing on the structural advancement of the endometrium and myometrium at birth. This study analyzed 60 fetal specimens aged 28–63 days post-conception using light, scanning, and transmission electron microscopy. Research has shown that tissue differentiation in the wall of the domestic cat’s uterine horns begins in the mid-prenatal period, i.e., around day 33 of gestation. The formation of the endometrium and myometrium in the uterine horns begins slightly later, i.e., between 42 and 51 days post-conception, and continues until the end of pregnancy. By birth, the uterine wall possesses all the layers found in adults, though full maturation, including gland development and epithelial remodeling, occurs postnatally. These findings provide critical insights into feline reproductive physiology and have broader implications for comparative embryology and veterinary diagnostics. Given the domestic cat’s significance in human society, understanding its reproductive development enhances veterinary care and supports future research on developmental disorders across species.

## 1. Introduction

The uterus of mammals, including the uterine tubes and the cranial vagina, develops from paired paramesonephric (Müllerian) ducts [1,2,3]. It is widely accepted that these ducts originate from the cephalolateral invagination of the coelomic epithelium to the upper regions of the mesonephroi, followed by the caudal elongation of the resulting epithelial tubes [3,4,5,6].

In domestic animals, prenatal uterine development follows variable patterns, as the uterine and uterovaginal segments of the paramesonephric ducts may fuse completely, partially, or minimally [2]. In the domestic cat, the long uterine horns derive from the middle uterine segments of the paramesonephric ducts, whereas the short uterine body develops from the uterovaginal canal [6].

Various aspects of uterine development have been investigated in rodents, domestic animals, and humans. The histogenesis of the uterine wall has been described in mice and rats [7], sheep [7,8,9], cattle [9], pigs [7,10], and humans [11,12,13]. However, studies on uterine development in carnivores, including cats, remain limited. Using light microscopy, Inomata et al. [14] described the development of the paramesonephric and mesonephric ducts in male and female domestic cats with crown–rump length (CRL) ranging from 0.8 to 10.5 cm, aiming to identify the timing of sexual differentiation of these structures. Additionally, Merlo et al. [15] characterized the development of uterine glands in domestic cats from late gestation to puberty using light microscopy.

In our previous morphological and three-dimensional reconstruction study of domestic cat fetuses, we identified the stages of reproductive system development and determined the spatial and temporal differentiation of the paramesonephric duct rudiments into uterine tubes and uterine horns [6]. We found that the first stage of feline internal reproductive organ development lasts from day 26 to 44 p.c., and it involves the formation of the tubal and uterine segments of the paramesonephric ducts. The second stage continues until the end of the prenatal period and entails the histodifferentiation of the paramesonephric duct wall, following the structural patterns assigned to the uterine tubes and the uterus. These studies also demonstrated changes in the topographical relationships between the developing female reproductive tract, the ovaries, and the mesonephros and indicated conversions in the shape of the paramesonephric ducts, which, after day 54 p.c., transform into the uterus, shifting from a U-shaped to a W-shaped configuration. The final shape and topography of the uterus in the domestic cat are established by the third month of postnatal development when the uterine horns straighten and, together with the uterine body, form a V-shaped structure within the abdominal cavity [6].

In another study, we described the sequence of ultrastructural changes in the endometrial epithelium of the paramesonephric ducts and developing uterine horns in domestic cats [16]. The study identified two primary stages in uterine epithelium development. The first stage, lasting until day 42 p.c., involves the formation of the pseudostratified epithelium of the paramesonephric duct. The second stage concerns the development of the endometrial epithelium, which remains pseudostratified with variable height by the end of the prenatal period and only transforms into simple columnar epithelium after birth [16]. The postnatal development of the domestic cat’s uterine horns has also been documented, with a particular focus on changes in the endometrium and its maturation necessary for reproductive function [17].

The present study aimed to describe the prenatal histodifferentiation of the uterine wall in domestic cats during the development of the uterine segments of the paramesonephric ducts and the uterine horns, with particular emphasis on the structural maturity of the endometrium and myometrium at birth. The findings are discussed in the context of both the prenatal development of the uterine tubes and postnatal microstructural changes in the uterine horns. Expanding knowledge of uterine development in domestic cats—one of the most widespread and socially integrated companion species—is essential for advancing our understanding of feline reproductive physiology. This study contributes valuable new insights into uterine wall histodifferentiation, enriching the field of veterinary science and comparative embryology and offering a foundation for future cross-species research. Moreover, a better understanding of embryonic development in the feline reproductive system has practical implications for veterinary diagnostics, particularly in identifying developmental abnormalities.

## 2. Materials and Methods

Studies were conducted on uteri collected from 60 euthanized fetuses of domestic cats aged 28–63 days p.c. (post-conception). In fetuses older than 33 days p.c., euthanasia was performed using Morbital (pentobarbital sodium and pentobarbitone; Biowet, Puławy, Poland). The fetuses were obtained postmortem from wild living female cats subjected to surgical ovariohysterectomy for clinical reasons, provided by veterinary clinics in Poznan (Poland). The age of fetuses was estimated according to the crown–rump length (CRL) and growth curve of Evans and Sack [18]. The fetuses were measured from the tip of the head to the root of the tail. The measurement was taken along the natural curve of the spine using a string. This study followed the guidelines of Directive 2010/63/EU on the protection of animals used for scientific purposes. According to Polish law, the experiments conducted within this study did not require approval from the Local Ethical Committee for Experiments on Animals.

For light microscopy (LM), cross-sections of the uterine horns were collected from 30 fetuses aged 28, 33, 42, 51, and 63 days p.c. Tissues were fixed for 24–48 h in 4% formalin or Bouin solution, dehydrated for 30 min in a graded series of ethanol (50–96%), transferred to methyl benzoate, and then placed for one hour at 60 °C in Paraplast^®^. Afterward, the samples were embedded in Paraplast blocks, and the serial sections of 4.5 µm were cut using an RM 2055 microtome (LEICA, Wetzlar, Germany). To remove Paraplast, slides were placed for 15 min in xylene and then stained with the Masson–Goldner trichrome method and with Orcein or Orcein–Picroindigocarmin staining [19,20]. Observations of the histological slides were made using an Axioskop 2 plus microscope (ZEISS, Oberkochen, Germany) and KS400 image analysis system v. 3.0 (ZEISS, Oberkochen, Germany). The morphometric analysis was performed using MultiScan v. 18.03 software (CSS, Warszawa, Poland). For each developmental stage (i.e., days 28, 33, 42, 51, and 63 p.c.), six individuals were selected for analysis. To estimate the thickness of the wall and its individual layers, 30 measurements were taken on each day of the study, while 42 measurements were taken to determine the height of the epithelium. The larger number of measurements taken from the uterine horn epithelium was due to its variable height. The morphometric data are presented in Table 1.

For scanning electron microscopy (SEM), cross-sections of the uterine horns were collected from 24 fetuses aged between 28 and 63 days p.c. The Paraplast^®^ sections of 10–20 µm were used for the SEM observations. The slides were placed for 15 min in xylene and then dehydrated in 96% ethanol for 2 min. Then, the slides were dried at room temperature, mounted on aluminum stubs covered with carbon tape, and then gold-sputtered for 1 min in Sputter Coater S 150B (BOC Edwards, Crawley, England). The preparations were observed and documented with a scanning electron microscope LEO 435 VP (ZEISS, Oberkochen, Germany) at an accelerating voltage of 10–15 kV.

For the transmission electron microscopy (TEM), cross-sections of the uterine horns were taken from 6 fetuses aged 33, 42, 55, and 63 days p.c. The tissues were fixed for an hour in 2.5% glutaraldehyde in cacodylate buffer (pH = 7.2) at 0–4 °C and for 40 min in 4% osmium tetroxide solution in cacodylate buffer (pH = 7.2) at 4 °C. After fixation, samples were dehydrated for 15 min in a graded series of ethanol (30–96%) and placed for 5 min in a 1:1 mixture of ethanol–propylene oxide and for 5 min in propylene oxide. Dehydrated tissues were incubated for 12 h in three changes of the solution of propylene oxide with Epon 812 (proportions: 3:1, 1:1, 1:3) and then embedded in Epon 812 epoxy resin with 2% DMP–30. The polymerization of the resin was conducted at 37 °C, 45 °C, and 60 °C. Epon blocks were cut into 2.5 µm semithin sections and stained with 1% toluidine blue. The ultracut microtome (LEICA, Wetzlar, Germany) was used to cut ultrathin sections of 70 nm, which were then counterstained with 2% aqueous uranyl acetate and lead citrate. The electron micrographs were observed and documented under the transmission electron microscope JEM1200 EX II (JEOL, Tokyo, Japan).

## 3. Results

### 3.1. Day 28 p.c. (CRL of 28 mm)

**Figure 1:**
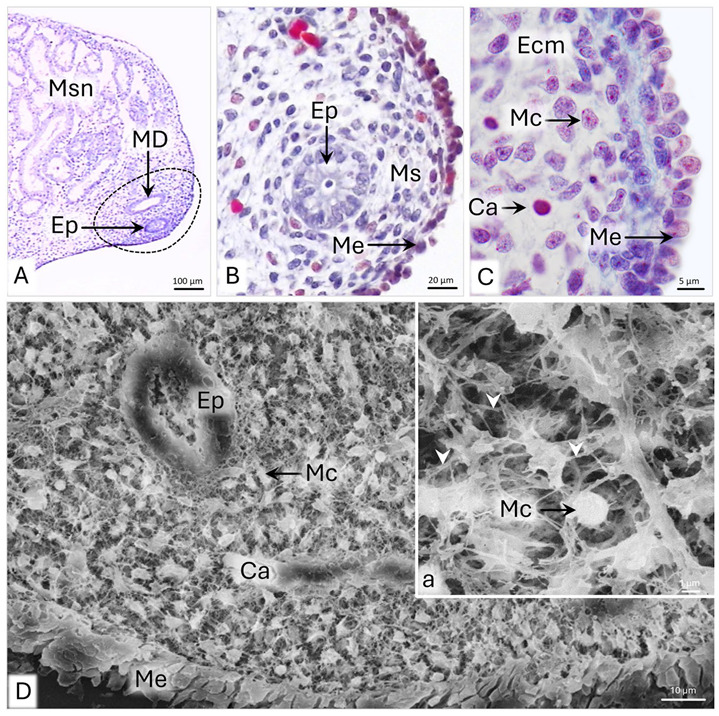
Domestic cat. Day 28 p.c. (**A**,**B**): Cross-section of the uterine segment of the paramesonephric duct (uPD). (**C**) Magnification of the uPD wall; LM: Masson–Goldner staining. (**D**): Cross-section of the wall of uPD; a—magnification of the mesenchymal cells. White arrowheads indicate protrusions of mesenchymal cells, SEM. Ca—capillary; Ep—epithelium; Ecm—extracellular matrix; Msn—mesonephros; Mc—mesenchymal cell; MD—mesonephric duct; Me—mesothelium; Ms—mesenchyme.

On day 28 p.c., the wall of the uterine segment of the paramesonephric duct (uPD) is composed of a single-layered simple columnar epithelium, encircled by the mesenchyme lined with the mesothelial epithelium (Figure 1A,B). The mean thickness of the wall is about 49 µm (Table 1). The epithelium of the duct lies on a continuous, flat basement membrane and is composed of 17 µm high cylindrical cells with round cell nuclei (Figure 1B and Table 1). The approximately 5 µm wide mesenchymal cells possess 1–3 µm long cytoplasmic protrusions by which they contact each other and oval cell nuclei, occupying up to 90% of the cell’s cytoplasm (Figure 1B,D). The extracellular matrix of the mesenchyme is homogeneous and, on histological slides, becomes bluish in aniline blue staining, which shows up especially abundantly under the mesothelium (Figure 1C). Single, 10 µm wide capillaries are observed in the periphery of the uPD wall (Figure 1B). The outer part of the wall is lined by the mesothelial epithelium, composed of cuboidal cells, which protrude into the abdominal cavity (Figure 1B).

### 3.2. Day 33 p.c. (CRL of 40 mm)

**Figure 2:**
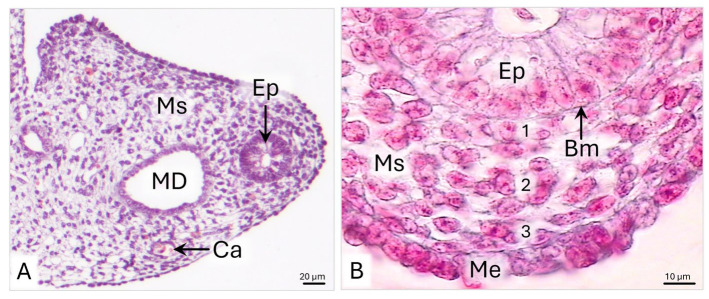
Domestic cat. Day 33 p.c. (**A**): Cross-section of the uterine segment of the paramesonephric duct (uPD). (**B**): Magnification of the uPD wall, presenting subepithelial (1), middle (2), and outer (3) parts of the wall; LM: Masson–Goldner staining. Bm—basement membrane; Ca—capillary; Ep—epithelium; MD—mesonephric duct; Me—mesothelium; Ms—mesenchyme.

On day 33 p.c., the wall of the uterine segment of the paramesonephric duct (uPD) is composed of pseudostratified epithelium and mesenchyme covered by mesothelial epithelium (Figure 2A,B). The mean thickness of the wall is about 58.9 µm (Table 1). The pseudostratified epithelium of approximately 19 µm in height is characterized by a flat surface (Figure 2B and Table 1).

**Figure 3:**
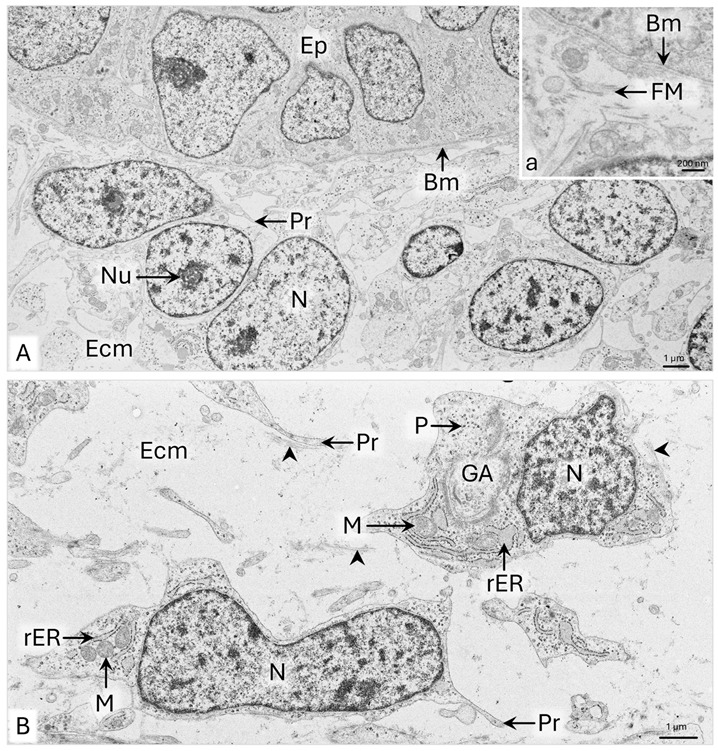
Domestic cat. Day 33 p.c. (**A**): Ultrastructure of the mesenchymal cells in the subepithelial part of the wall of a uterine segment of the paramesonephric duct (uPD); a—magnification of the area lying beneath the basement membrane. (**B**): Ultrastructure of the mesenchymal cells in the middle of the uPD wall. Arrowheads indicate fibers deposited around the mesenchymal cells, TEM. Bm—basement membrane; Ep—epithelium; Ecm—extracellular matrix; FM—fibrillar material; GA—Golgi apparatus; M—mitochondria; N—cell nucleus; Nu—nucleoli; P—polysome; Pr—cytoplasmic protrusion; rER—rough endoplasmic reticulum.

The mesenchyme surrounding the epithelial tubule of the paramesonephric duct has 7–10 layers of cells (Figure 2B). In the subepithelial part of the wall, the mesenchymal cells are approximately 10 µm long and possess large oval-shaped cell nuclei with a diameter of about 8 µm and single nucleoli (Figure 3A). Thin protrusions of these cells are 1–3 µm long. In the cytoplasm, single cisternae of rough endoplasmic reticulum (rER), mitochondria with a 400–800 nm diameter, and scattered polysomes of five to six ribosomes are observed (Figure 3A). Fine fibrillar material is deposited on the surface of mesenchymal cells, especially in the subepithelial area beneath the basement membrane (Figure 3A-a).

In the middle of the uPD wall, mesenchymal cells are characterized by elongated cell nuclei with evenly dispersed chromatin without nucleoli and 2 µm long cytoplasmic protrusions (Figure 3B). These cells contain few cisternae of rER, many polysomes, a Golgi apparatus, and 500–800 nm wide mitochondria. The elongated cisternae of rER are placed along the mitochondria or encircle them (Figure 3B). On the surface of the cell’s membrane, locally, 1–1.5 µm long collagen fibrils are visible (Figure 3B).

**Figure 4:**
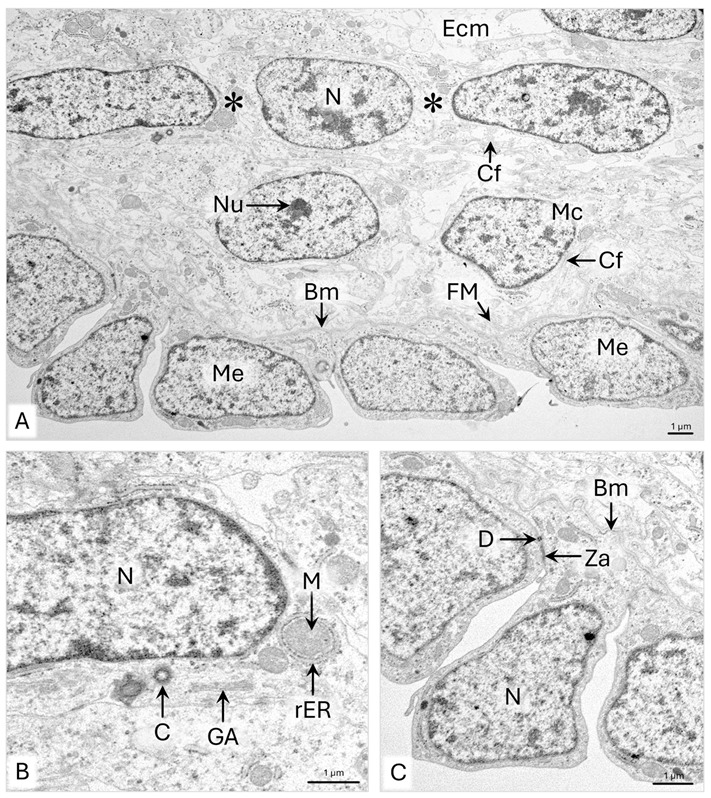
Domestic cat. Day 33 p.c. (**A**): Ultrastructure of the mesenchymal cells in the outer (subserosal) part of the uterine segment of the paramesonephric duct (uPD) wall. Asterisks indicate a row of elongated mesenchymal cells. (**B**): Magnification of the elongated mesenchymal cell. (**C**): Ultrastructure of the mesothelium of the uPD, TEM. Bm—basement membrane; C—centrosome; Cf—collagen fibers; D—desmosome; Ecm—extracellular matrix; FM—fibrillar material; GA—Golgi apparatus; M—mitochondria; Mc—mesenchymal cell; Me—mesothelium; N—cell nucleus; Nu—nucleoli; rER—rough endoplasmic reticulum; Za—zonula adherens.

In the outer part of the uPD wall, the elongated, closely packed mesenchymal cells form one to two rows of cells with a circular arrangement (Figure 4A). These cells are approximately 18 µm long and possess elongated cell nuclei with single nucleoli (Figure 4A). Their cytoplasm contains single cisternae of rER, Golgi apparatus, 400–900 nm wide mitochondria, and well-visible centrosomes in the perinuclear area (Figure 4B). The extracellular matrix of the outer part of the wall contains approximately 700 nm wide bundles of collagen fibers. Single fibrils and fine fibrillar material are also located around the cells and beneath the mesothelium (Figure 4A). Approximately 20 µm wide capillaries are observed in the outer part of the wall up to the mesenteric part of the uPD (Figure 2A).

The mesothelial epithelium of the duct is composed of cuboidal cells with an average height of 4.5–9 µm (Figure 2B and Figure 4A). On electron micrographs, a slightly undulated basement membrane of mesothelium is visible. (Figure 4C). The mesothelial cells are connected only in the basal part, where zonula adherens and desmosomes are present, so the cells protrude into the abdominal cavity (Figure 4C).

### 3.3. Day 42 p.c. (CRL of 77 mm)

**Figure 5:**
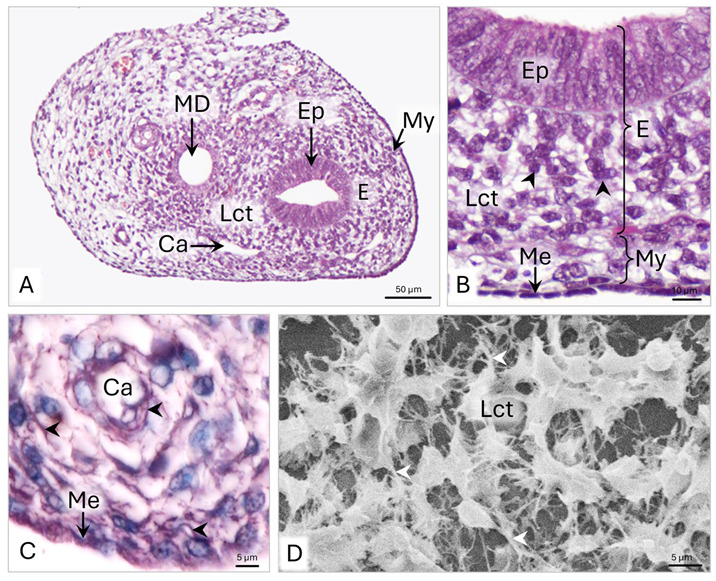
Domestic cat. Day 42 p.c. (**A**): Cross-section of the uterine segment of the paramesonephric duct (uPD). (**B**): Magnification of the uPD wall. Black arrowheads indicate columns of fibroblasts; LM: Masson–Goldner staining. (**C**): Magnification of the uPD submesothelial area. Arrowheads indicate the elastic fibers under the mesothelial epithelium; LM: Orcein–Picroindigocarmine staining. (**D**): Magnification of the loose connective tissue cells in the wall of uPD. White arrowheads indicate the cytoplasmic protrusions of the fibroblasts, SEM. Ca—capillary; E—endometrium; Ep—epithelium; Lct—loose connective tissue; Me—mesothelium; MD—mesonephric duct; My—myometrium.

On day 42 p.c., the wall of the uterine segment of the paramesonephric duct (uPD) consists of endometrium composed of pseudostratified epithelium, loose connective tissue lamina propria, and myometrium covered with mesothelium (Figure 5A,B). The average thickness of the wall is 95.8 µm (Table 1). The epithelium is approximately 29 µm high and has a flat surface (Figure 5B and Table 1).

**Figure 6:**
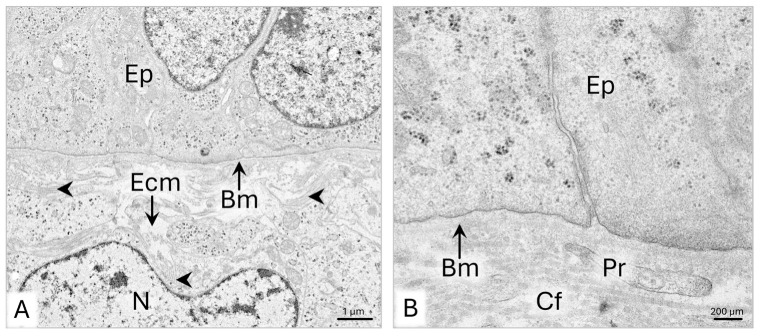
Domestic cat. Day 42 p.c. (**A**): Ultrastructure of the subepithelial part of the wall of the uterine segment of the paramesonephric duct (uPD). Arrowheads indicate bundles of collagen fibers. (**B**): Magnification of the area beneath the basement membrane of uPD epithelium, TEM. Bm—basement membrane; Cf—collagen fibers; Ecm—extracellular matrix; Ep—epithelium; N—cell nucleus; Pr—cytoplasmic protrusion.

**Figure 7:**
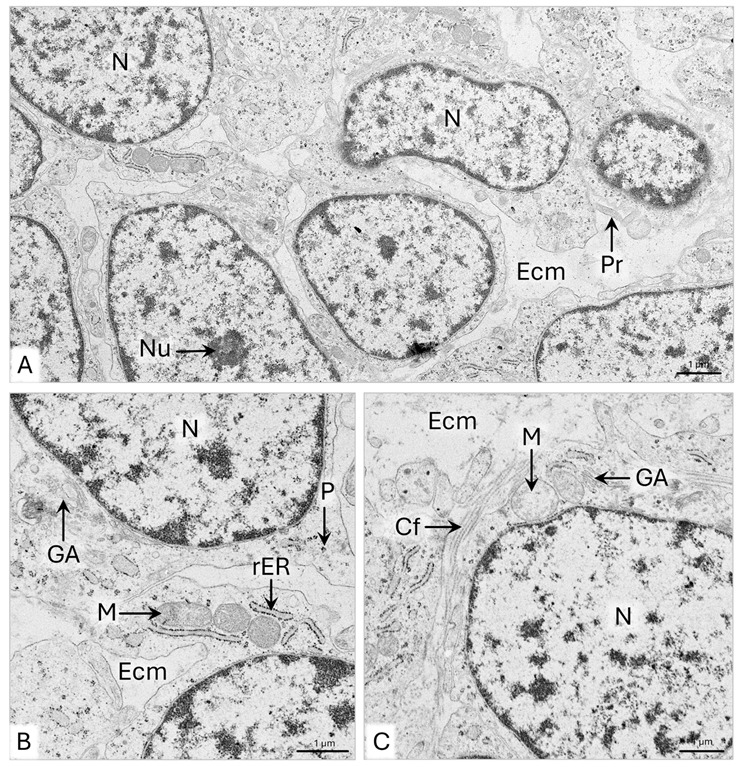
Domestic cat. Day 42 p.c. (**A**): Ultrastructure of the endometrial fibroblasts in the wall of the uterine segment of paramesonephric duct (uPD). (**B**,**C**): Magnification of fibroblasts in the middle of uPD wall, TEM. Cf—collagen fibers; Ecm—extracellular matrix; GA—Golgi apparatus; M—mitochondria; N—cell nucleus; Nu—nucleoli; P—polysome; Pr—cytoplasmic protrusion; rER—rough endoplasmic reticulum.

The endometrium is 39 µm wide and comprises 7 to 10 layers of fibroblasts (Figure 5B). The 10–15 µm long fibroblasts are organized in columns with a radial arrangement in relation to the uterine epithelium (Figure 5D). The cells have 2–4 µm long cytoplasmic protrusions and oval nuclei, with single nucleoli and clusters of condensed chromatin distributed under the nuclear envelope (Figure 6A and Figure 7A). In their cytoplasm, elongated cisternae of rER are observed with locally extended lumen, round or oval-shaped mitochondria with a length of 0.5–1 µm, and a Golgi apparatus (Figure 7B). Around their cell membrane occur single, maximum 1 µm long collagen fibrils and 200–400 nm wide bundles of collagen fibers (Figure 7C). A particularly high amount of collagen fibers, forming 400–600 nm wide bundles, is observed directly under the basement membrane of the pseudostratified epithelium (Figure 6B). Between the loosely dispersed fibroblasts, 10–20 µm wide circumferential capillaries occur in the middle of the wall.

The 16.6 µm wide myometrium consists of three to four rows of fibroblasts and myocytes with a mainly circular arrangement (Figure 5B). The mesothelial epithelium is simple cuboidal, with an average height of 5 µm. The mesothelial cells bulge slightly into the abdominal lumen (Figure 5B). On LM slides stained with aniline blue and orcein, collagen fibers and elastic fibers under the mesothelium are noted (Figure 5C).

### 3.4. Day 51 p.c. (CRL of 110 mm)

**Figure 8:**
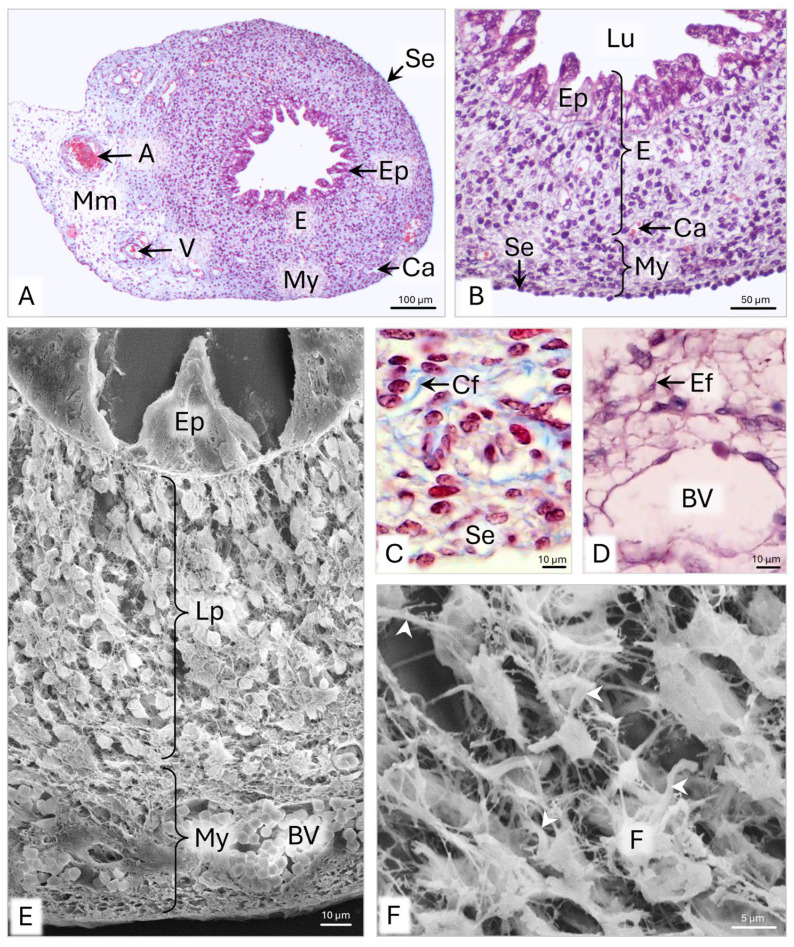
Domestic cat. Day 51 p.c. (**A**,**B**): Cross-section of the uterine horn (UH). (**C**): Magnification of the UH myometrium with collagen fibers; LM: Masson–Goldner staining. (**D**): Magnification of the myometrium with elastic fibers; LM: Orcein staining. (**E**): Cross-section of the UH wall. (**F**): Arrangement of fibroblasts and collagen fibers (white arrowheads) in the endometrial lamina propria. SEM. A—artery; BV—blood vessel; Ca—capillary; Cf—collagen fibers; E—endometrium; Ef—elastic fiber; Ep—epithelium; F—fibroblast; Lp—endometrial lamina propria; Lu—lumen; Mm—mesometrium; My—myometrium; Se—serosa; V—vein.

On day 51 p.c., the developing uterine horn (UH) wall consists of an endometrium lined by the pseudostratified epithelium and the myometrium, covered by a serosa (Figure 8A,B). The UH wall is approximately 188 µm thick (Table 1). The endometrial epithelium averages 41 µm in height and is characterized by a variable height (Figure 8B and Table 1).

**Figure 9:**
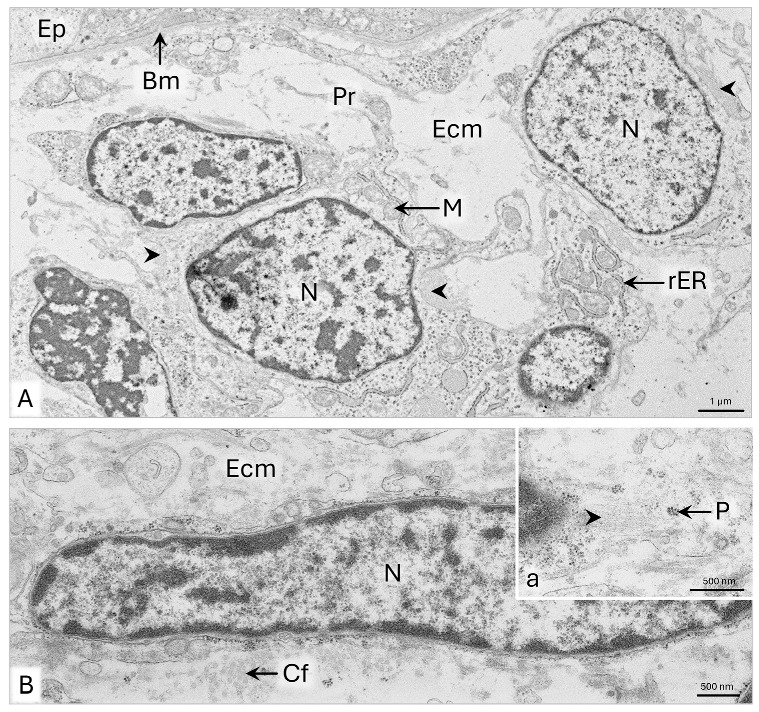
Domestic cat. Day 51 p.c. (**A**): Ultrastructure of the fibroblasts in the uterine horn (UH) endometrium. Arrowheads indicate the bundles of collagen fibers between fibroblasts. (**B**): Ultrastructure of the smooth muscle cell (sMC); a—magnification of the sMC cytoplasm, with bundles of thin filaments (arrowheads), TEM. Bm—basement membrane; Cf—collagen fibers; Ecm—extracellular matrix; Ep—epithelium; M—mitochondria; N—cell nucleus; P—polysome; Pr—cytoplasmic protrusion; rER—rough endoplasmic reticulum.

The 117 µm thick endometrial lamina propria consists of approximately 15 fibroblast layers arranged in columns oriented perpendicularly or obliquely to the uterine horn epithelium (Figure 8B,E and Table 1). The fibroblasts are 10–12 µm long and possess approximately 3 µm long cytoplasmic protrusions (Figure 8F). The cytoplasm of the fibroblasts contains numerous polysomes with an even distribution, a Golgi apparatus, and mitochondria as large as 1 µm, encircled by an elongated rER cisternae (Figure 9A). Throughout the endometrial extracellular matrix, bundles of collagen fibers as long as approximately 2 µm and as wide as 1.2 µm are observed around the cells (Figure 9A).

The myometrium of the uterine horn is about 41.5 µm wide and comprises approximately seven cell layers with a circular arrangement (Figure 8B and Table 1). Among the cells are fibroblasts and elongated smooth muscle cells. The smooth muscle cells are about 10–15 µm long and 1–1.5 µm wide. Electron micrographs revealed a strongly elongated cell nucleus, filling most of the cell volume (Figure 9B). A little cytoplasm with free ribosomes extends along the cell nucleus. The narrow ends of cells contain polysomes composed of six to eight ribosomes and clusters of thin filaments, forming 400 nm wide bundles (Figure 9B-a). Collagen fibers with a circular or longitudinal arrangement are observed between the fibroblasts and smooth muscle cells (Figure 9B). Within the myometrium, the 20–40 µm wide blood vessels have a circumferential arrangement (Figure 8A,E). Outside the myometrium lies the serosa, consisting of simple cuboidal epithelium, lying on loose connective tissue. The epithelium comprises a single layer of cells that protrudes into the abdominal cavity (Figure 8B).

On LM images, aniline blue staining of collagen fibers is observed under the uterine horn lining and serous epithelium, as well as in the lower part of the myometrium (Figure 8C). Orcein staining shows the presence of elastic fibers in the myometrium and the wall of blood vessels (Figure 8D).

### 3.5. Day 63 p.c. (CRL of 150 mm)

**Figure 10:**
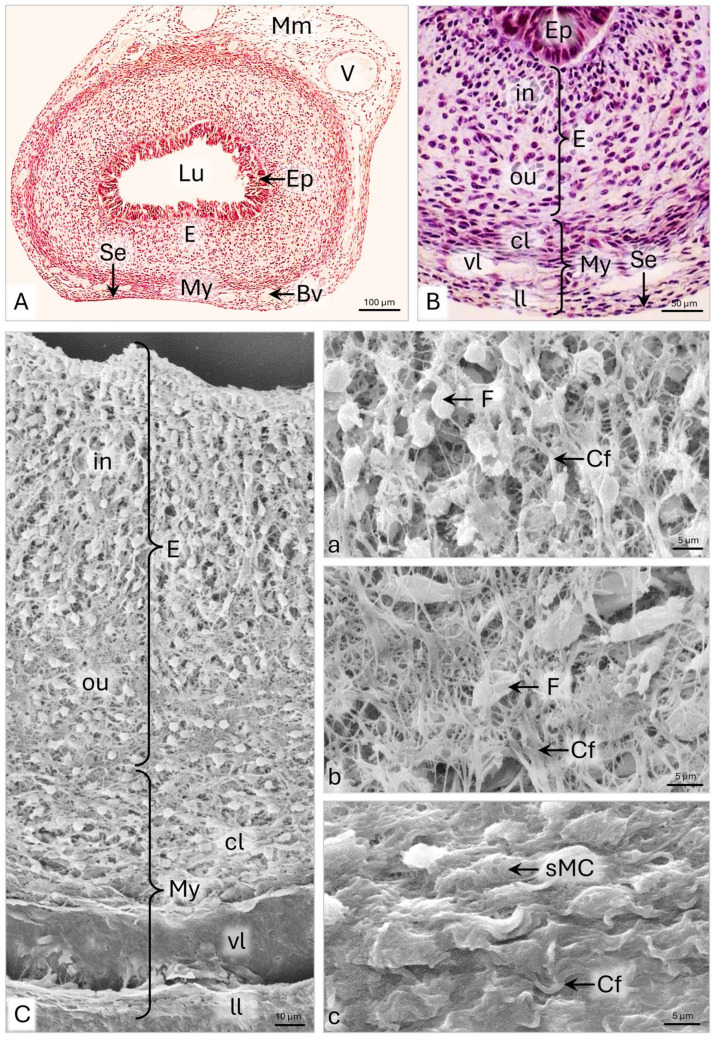
Domestic cat. Day 63 p.c. (**A**,**B**): Cross-section of the uterine horn (UH); LM: Masson–Goldner staining. (**C**): Cross-section of the UH wall; a—magnification of the inner layer of the endometrium; b—magnification of the outer layer of the endometrium; c—magnification of the circular layer of the myometrium, SEM. Bv—blood vessel; Cf—collagen fibers; cl—circular layer of myometrium; E—endometrium; Ep—epithelium; F—fibroblast; in—inner layer of the endometrium;ll—longitudinal layer of myometrium; Lu—lumen; Mm—mesometrium; My—myometrium; ou—outer layer of the endometrium; Se—serosa; sMC—smooth muscle cel; vl –vascular layer of myometrium; V—vein.

On day 63 p.c., the uterine wall of the domestic cat measures approximately 316 µm in thickness and is composed of endometrium, myometrium, and serosa with a broad subserosal area in the mesenteric part of the wall (Figure 10A,B and Table 1). The endometrial epithelium remains pseudostratified with a variable height, ranging from 36 to 57 µm (Figure 10A and Table 1).

The lamina propria of the endometrium is approximately 163 µm thick, and on its cross-section, the inner and outer layers are clearly visible (Figure 10B and Table 1). Fibroblasts of the inner layer have a columnar arrangement; in the endometrium’s outer layer, they appear scattered (Figure 10B,C). Analysis of the endometrial cell distribution showed that on a cross-section of 1000 µm^2^, there are approximately 18–19 cells in the inner layer and approximately 11–12 cells in the outer layer.

**Figure 11:**
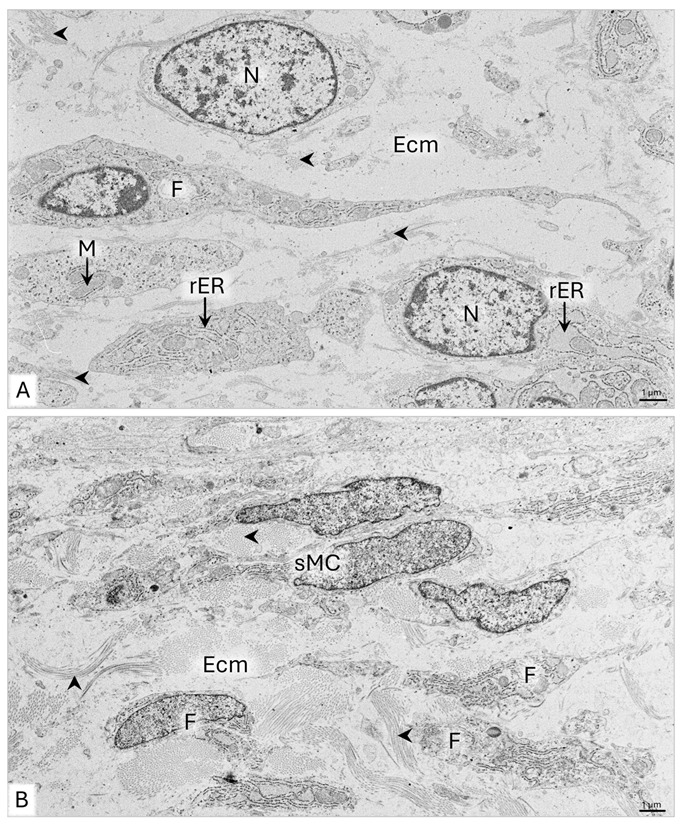
Domestic cat. Day 63 p.c. (**A**): Ultrastructure of the fibroblasts in the inner layer of the uterine horn endometrium. Arrowheads indicate bundles of collagen fibers. (**B**): Ultrastructure of the smooth muscle cells (sMC) and fibroblasts (F) in the circular layer of the myometrium. Arrowheads indicate bundles of collagen fibers. TEM. Ecm—extracellular matrix; M—mitochondria; N—cell nucleus; rER—rough endoplasmic reticulum.

TEM showed that endometrial fibroblasts are spindle-shaped cells, approximately 20 μm in length, with cytoplasmic protrusions 1–3 μm long and 200–400 nm wide (Figure 11A). They have large oval cell nuclei with clusters of condensed chromatin under the nuclear envelope (Figure 11A). Numerous elongated and locally widened cisternae of rER, evenly distributed polysomes, and the Golgi apparatus are noted in the perinuclear area and the cytoplasmic protrusions. The mitochondria are round, oval, or elongated, ranging from 400 nm to 1.7 µm in length (Figure 11A). Collagen fibers, observed between fibroblasts, are up to 3 µm long and form bundles with a width of 0.5–1.2 µm (Figure 11A). SEM observations revealed that the arrangement of the thick bundles of collagen fibers throughout the endometrial lamina propria corresponds to the cell distribution (Figure 10C). In the inner layer of the endometrium, the collagen fiber bundles are arranged along the columns of cells (Figure 10C-a). In the outer layer, they are scattered, forming a dense network of interconnected collagen bands (Figure 10C-b).

The myometrium of the uterine horn is, on average, 95.5 µm thick and consists of approximately 15 cell layers, among which fibroblasts and myocytes occur (Figure 10B and Table 1). The inner circular, intermediate vascular, and outer longitudinal layers can be distinguished. In the wide circular layer, the smooth muscle cells have a tight arrangement, with fibroblasts and strands of thick bundles of collagen fibers scattered between them (Figure 10B and Figure 10C-c). The thin longitudinal layer comprises loosely arranged myocytes and fibroblasts (Figure 10B). Electron micrographs reveal that the smooth muscle cells are elongated and have irregularly shaped or corrugated cell nuclei with evenly dispersed chromatin (Figure 11B). The cell nucleus is surrounded by a narrow cytoplasm limited by a sarcolemma (Figure 11B). A particularly high density of collagen fibers is observed in the extracellular matrix, forming 6 µm thick bundles positioned in many directions (Figure 11B). Serosa is composed of a simple squamous epithelium and a thin layer of loose connective tissue (Figure 10A).

## 4. Discussion

The results presented in this study demonstrate the sequence of structural changes in the wall of the developing uterine horns, from the primordial uterine segments of the paramesonephric ducts to the end of the prenatal period. Observations using light microscopy (LM), scanning electron microscopy (SEM), and transmission electron microscopy (TEM) enabled us to describe the histodifferentiation of the uterine wall from mesenchyme into the endometrial loose connective tissue and the myometrial smooth muscle tissue.

The differentiation of uterine mesenchyme into endometrial connective tissue and myometrial smooth muscle begins between days 28 and 33 p.c. As revealed by our observations, on day 28 p.c., the uterine horns of the domestic cat appear as rudimentary uterine segments of the paramesonephric ducts, characterized by an embryonic wall composed of a simple epithelium surrounded by mesenchyme. At this stage, the only indicator of mesenchymal cell activity is the bluish staining of the amorphous extracellular matrix in histological slides, suggesting the secretion of proteoglycans. By day 33 p.c., the wall of the uPD loses its embryonic features: the epithelium becomes pseudostratified, and the mesenchymal cells differentiate into fibroblasts. In addition to proteoglycans, these cells secrete fine fibrous material into the intercellular space, particularly accumulating beneath the epithelial basement membrane. Collagen fibrils are also deposited on the surfaces of fibroblasts. In the peripheral region of the wall, elongated mesenchymal cells assume a circular arrangement and become precursors of future myoblasts.

According to the literature, early mesenchymal differentiation in the uterine wall is characteristic of humans and livestock, species with relatively long gestation periods [7,8,12]. In humans, fibroblasts and smooth muscle cells differentiate by the 20th week of gestation [7,12,21], while in sheep, this process begins around day 55 of prenatal development [8]. In contrast, in rodents, the uterine wall mesenchyme remains undifferentiated until the neonatal period [7,22].

In the domestic cat, the development of loose connective and smooth muscle tissue in the uPD, beginning before day 33 p.c., leads to the formation of the endometrium and myometrium. During endometrial development, the epithelial structure changes, and fibroblasts organize into patterns that correspond to the future basal and functional layers of the mucosal lamina propria. Between days 42 and 51 p.c., the uterine epithelium becomes pseudostratified and displays variable height. Over the prenatal development, this epithelium increases in height nearly threefold, and by the end of the prenatal period, its surface exhibits marked undulations. Previous studies have shown that the simple columnar structure of the uterine epithelium in domestic cats is established approximately one month after birth, coinciding with the appearance of uterine gland primordia in the mucosal lamina propria [17].

The formation of the endometrial lamina propria begins after day 33 p.c., when fibroblasts arrange into columns and align radially around the uterine epithelium. Initially, the alignment of fibroblast columns is perpendicular to the epithelium, shifting to an oblique orientation by day 51 p.c. The distinct division of the endometrium into basal and functional layers becomes apparent toward the end of the prenatal period, evidenced by differences in fibroblast density and spatial distribution. The inner functional layer contains fibroblasts arranged in columns and has up to 60% more cells than the outer layer, where fibroblasts are irregularly distributed.

A double-layered endometrium during prenatal development has also been reported in sheep and humans [7,8,12]. In contrast, studies in pigs have shown that the endometrium does not divide into layers until after birth; distinct subepithelial tissue areas become apparent only about a week postpartum [7,10].

SEM observations revealed that, in the domestic cat, collagen fibers progressively accumulate in the extracellular matrix, aligning with the cellular organization of the mucosal layers. By the end of the prenatal period, thick collagen fiber bundles reflect a columnar arrangement of fibroblasts in the inner layer and a more irregular pattern in the outer layer. Our previous research on postnatal uterine development in domestic cats showed that this specific organization of fibroblasts and collagen bundles in the functional layer of the endometrium is maintained for the first 3 to 4 weeks after birth. However, as uterine glands begin to invade the lamina propria, this arrangement gradually becomes less distinct [17]. These findings suggest that the columnar organization of fibroblasts, along with associated collagen fibers and capillaries, may provide structural guidance for developing glandular tubules. This arrangement likely offers both a stable connective tissue framework and effective vascular support.

Morphometric analysis revealed that between days 42 and 63 p.c., the thickness of the endometrial lamina propria in the domestic cat increases more than fourfold. Notably, the endometrium remains smooth until the end of the prenatal period. Mucosal folds begin to form 3 to 4 weeks after birth and continue developing at least until the third month of life. This process is likely related to the need for creating sufficient space in the functional layer of the endometrium to accommodate the expanding uterine glands [17].

The formation of myometrial smooth muscle tissue begins on day 33 p.c. with the differentiation of the first myoblasts, which appear in a circular arrangement at the periphery of the uPD wall. By day 42 p.c., the myometrium becomes recognizable. At this stage, it is more than twice as thin as the endometrial lamina propria and consists of several rows of circularly arranged smooth muscle cells interspersed with fibroblasts. Subsequent development of the myometrium involves an increase in the number of myocyte layers. By the end of the prenatal period, the myometrium undergoes nearly a sixfold increase in thickness and occupies approximately one-third of the total uterine wall.

The characteristic microstructure of the myometrium, comprising three distinct layers, can be identified in the domestic cat at the end of the prenatal period. Between days 51 and 63 p.c., the myometrium divides into sublayers; however, their relative proportions differ substantially from those observed in the adult uterus. Just prior to birth, the predominant layer is the inner circular muscle layer, composed of several rows of smooth muscle cells. In contrast, the outer longitudinal layer and the vascular interlayer remain poorly developed. As described in our previous work, postnatal development of the myometrium in the domestic cat involves a progressive thickening of each layer, with particularly rapid growth of the outer longitudinal layer. In adult specimens, this layer is approximately 40% thicker than the inner circular layer [17]. Additionally, during the first three months after birth, the vascular layer expands and differentiates into a distinct intermediate myometrial layer [17].

The developmental pattern of the myometrium in the domestic cat resembles that observed in rodents, pigs, and humans, where differentiation of smooth muscle cells begins at the periphery of the uterine wall, with the inner circular layer forming first [10,21,22].

A comparative analysis of the prenatal development of the uterine horn and uterine tube walls in the domestic cat reveals both similarities and differences in the rate and pattern of tissue differentiation [23]. As both organs originate from a common primordium, they initially develop from a simple cylindrical epithelium surrounded by mesenchyme. A shared feature in their epithelial development is the transformation into a pseudostratified epithelium with variable height. In both organs, this pseudostratified form is temporary. In the uterine tube, it transforms into a simple ciliated columnar epithelium before birth, whereas in the uterus, this change occurs only postnatally [23].

Mesenchymal differentiation into loose connective tissue begins earlier in the uterine tube—around day 27 p.c.—than in the uterus. Moreover, the lamina propria of the uterine tube’s infundibulum begins to fold around day 40 p.c., while in the uterus, such folding occurs postnatally [23]. Smooth muscle cells also appear earlier in the uterine tube, specifically in the ampulla, around day 40 p.c. However, at birth, the uterine myometrium is more developed. In contrast, the tunica muscularis of the uterine tube still consists of only a single layer of circularly arranged smooth muscle cells [23].

Despite differences in the timing of tissue layer development, both the uterine tube and uterus remain physiologically inactive at birth, as indicated by their incomplete structural organization. Maturation and the acquisition of adult characteristics occur during the first months of life. Functional maturity of the uterine tube is marked by the presence of labyrinthine mucosal folds lined with ciliated epithelium, while in the uterus, it is evidenced by the formation of uterine glands and the thickening of the muscular layer.

## 5. Conclusions

The development of the uterine segments of the paramesonephric duct in the domestic cat follows a pattern typical of uterine morphogenesis. The stratification of the uterine wall begins between days 42 and 51 p.c., while the histodifferentiation of the uPD wall is initiated earlier, around day 33 p.c. As a result, the prenatal histogenesis of the uterine wall leads to the formation of all major structural layers observed in adult specimens. However, complete maturation—including the establishment of a simple columnar endometrial epithelium, the lamina propria folding, the formation of uterine glands, and the thickening of the outer and vascular myometrial layers—continues during the postnatal period.

## Figures and Tables

**Table 1:** Morphometric data of the wall of the developing uterine horn in the domestic cat. X—average value (µm), SD—standard deviation (µm), Min/Max—minimum/maximum value (µm), *n*—number of measurements for each stage of development. *—on days 28 and 33, p.c., the endometrium and myometrium are not distinguished.

STAGE OF DEVELOPMENT	Thickness of the Wall	Height of the Epithelium	Thickness of the Endometrial Lamina Propria	Thickness of the Myometrium
	X/SD Min/Max *n* = 30	X/SD Min/Max *n* = 42	X/SD Min/Max *n* = 30	X/SD Min/Max *n* = 30
Day 28 p.c.	48.9/6.6 40.1/58.4	17.8/2.9 12.6/21.4	*	*
Day 33 p.c.	58.9/12.7 38.5/78.9	19.1/3.2 13.9/22.9	*	*
Day 42 p.c.	95.8/16.1 73.8/136.6	29.3/4.9 24.7/41	39.1/4.9 31.2/49.7	16.6/4.4 7.5/25.5
Day 51 p.c.	187.9/25.8 156/241.1	41.1/5.5 34.2/52.2	117.7/12.1 83.1/136.8	41.5/2.4 37.1/45.6
Day 63 p.c.	316.5/13.7 292.1/330.9	47.7/5.8 36.9/57.4	162.9/30.9 111.9/212.2	95.5/11.5 69.4/107.4

## Data Availability

The original contributions presented in this study are included in the article. Further inquiries can be directed to the corresponding author.

## Abbreviations

The following abbreviations are used in this manuscript:

LM	Light microscopy;
TEM	Transmission electron microscopy;
SEM	Scanning electron microscopy;
uPD	Uterine segment of paramesonephric duct;
p.c.	Post-conception;
CRL	Crown–rump length;
rER	Rough endoplasmic reticulum;
UH	Uterine horn.
